# Why are some groups physically active and others not? A contrast group analysis in leisure settings

**DOI:** 10.1186/s12889-018-5283-2

**Published:** 2018-03-20

**Authors:** Ansgar Thiel, Hendrik K. Thedinga, Harald Barkhoff, Katrin Giel, Olesia Schweizer, Syra Thiel, Stephan Zipfel

**Affiliations:** 1 Institute of Sport Science, Eberhard Karls University of Tübingen, Tübingen, Germany; 2 Kinesiology and Exercise Sciences, University of Hawai’i, Hilo, USA; 3 Department of Psychosomatic Medicine and Psychotherapy, Medical University Hospital Tübingen, Tübingen, Germany

**Keywords:** Physical activity, Leisure time behaviour, Social groups, Obesity

## Abstract

**Background:**

This field study aims to investigate the determinants of physical activity of particularly active and inactive groups in their leisure environments. In order to consider the context in which physical activity occurs and to investigate whether cultural settings may influence physical activity, we conducted the study at pools in different cultural environments - Hawai’i and Germany.

**Methods:**

This study presents the quantitative data of a systematic (covert) participant observation. We recorded the physical activity of face-to-face interacting groups and analysed categories such as group size, estimated age of the group members, and verbal communication patterns. Total observation period was eight and a half months. In total, we observed 907 groups with the groups’ size varying between 2 and 8 members. For the general statistics, we accessed the significance of differences regarding the degree of physical activity dependent on the target variables. To better understand activity promoting and hindering mechanisms, special attention is given to the identification of contrasting factors that characterise groups which are very active or very inactive. For this, we conducted a classification tree analysis.

**Results:**

General statistical analysis shows that, overall, the most differentiating factor regarding physical activity was the body shape of the group members. While obese groups had the lowest average activity level, groups mainly consisting of people with an athletic body shape were the most physically active. Yet, classification tree analysis reveals that obesity itself does not necessarily determine physical inactivity levels. The identification of six contrasting clusters highlights that besides the body shape several factors interact regarding a group’s physical level. Such interacting factors were for example the degree of communication within the group, the gender- and age-related composition of the group, but also the equipment that had been brought to the beach/pool. Obese people were particularly inactive when they were members of frequently communicating, age-heterogeneous groups.

**Conclusions:**

Our study shows that several social factors determine the physical activity of very active and very inactive groups. In order to promote physical activity, future health initiatives should target these factors of a person’s network.

**Electronic supplementary material:**

The online version of this article (10.1186/s12889-018-5283-2) contains supplementary material, which is available to authorized users.

## Background

In the last ten years, public health research has paid increased attention to determinants of physical activity in order to fight the pandemic development of non–communicable diseases [1, 2]. Most studies in this regard have analysed socioeconomic correlates of physical activity, particularly the socioeconomic status [3–8]. The studies in unison report that a high socioeconomic position (including occupational position, income and educational level) positively correlates with a higher degree of physical activity in leisure time.

Further studies also analysed the effects of social networks on the willingness of an individual to be physically active. Ball et al. [9] note that physical activity is associated with individual social participation and neighbourhood interpersonal trust. Mc Neill, Kreuter, and Subramanian [10] identify five modifiable dimensions of the social environment that have an influence on the activity status of a person: firstly, social support and social networks; secondly, socioeconomic position and income inequality; thirdly, racial discrimination; fourthly, social cohesion and social capital; and fifthly, neighbourhood factors. Studies by Yu et al. [11] and Carlson et al. [12] confirm the impact of social networks and social support on the degree that people are physically active. However, in the latter study, this effect was particularly observable in participants with positive psychosocial attributes.

De la Haye and colleagues [13] were able to show that close friends in adolescent friendship networks have a significant influence on each other regarding a number of health related behaviours, especially the physical activity behaviour was “*found to be similar*” [13]. Carell, Hoekstra, and West [14] report effects of social contagion analogical to findings with regard to general health related behaviour [15]. They come to the conclusion that especially ‘poor’ fitness spreads on a person-to-person basis through a social network [14]. They conclude that their findings are “*consistent with the notion that people imitate the diet or exercise habits of their least fit friends, or use those friends’ fitness as a benchmark for their own*” [14]. Regarding beneficial outcomes on the willingness to be longer physically active, Scarapicchia and colleagues [16] note that it is particularly intrinsic motivation which seems to spread.

The existing data about behavioural and social factors of physical activity are mostly based on questionnaires or demographic statistics. Furthermore, most studies investigate psychosocial variables, such as attitudes, motivation, action planning, self-efficacy, or stress [3]. Observational studies are scarce. Therefore, there is still a lack of research and findings representing physical activity in ‘real life’, particularly in unsupervised contexts [17]. Finally, there is also a lack of research on which factors characterise people which are particularly prone to be physically active or inactive.

In order to close this research gap, we conducted a study on social determinants of physical activity in groups. According to Tajfel and Turner [18], we define a social group “*as a collection of individuals who perceive themselves to be members of the same social category, share some emotional involvement in this common definition of themselves, and achieve some degree of social consensus about the evaluation of their group and of their membership in it*” [18]. Groups are considered as a specific form of social environment that has a relevant impact on the behaviour of group members due to group’s specific behavioural rules, motives and norms [19]. Group membership influences behaviour because “*the way we perceive others will influence directly how we act towards them*” [19]. The underlying mechanism of this influence is imitation, as “*the demeanour, the presentation, the look, the size, and the physicality of bodies is automatically and deeply perceived and read as a text at an automatic and deep level of perception*” [20].

Our study was conceptualized as a multi-method participant observation study. In contrast to existing studies that are based on large data sets or self-reports, we decided to systematically observe physical activity-related behaviours “*in action*” [21] and thereby to consider the context in which physical activity occurs in order to fully understand activity promoting and hindering mechanisms. The basic idea of the study was to observe group behaviour in specific leisure settings, where the opportunity to be physically active is generally given. Following a methodological observation paradigm, we focused exclusively on face-to-face interacting social groups and their visible actions. Since cultural values have an influence on how people deal with their own body [22–24], we also wanted to analyse their impact on the occurrence of very active and inactive groups. Cultural representations form the individual perception of how individual behaviour and appearance in a certain social context should be [25, 26]. Stereotypical and normative body ideals as well as somatic practices therefore symbolise the socially ‘expected’ body appearance and activity. For this reason, we decided to conduct the research in two different countries, where we expect culturally given differences regarding social expectations towards body appearance: a more multi-cultural environment: Hawai’i, and a more mono-cultural environment: Germany. For example, in both Hawai’i and Germany, obese bodies are considered not to conform with the societal body ideal [27, 28]. However, while in Germany obese bodies are generally stigmatised as unhealthy and considered to be caused by a lack of discipline [28], overweight bodies in Hawai’i are also related to Hawai‘i’s cultural traditions “*that embrace food as an offering of gratitude, graciousness*” [29].

We have already published an article about the qualitative data of this study which focused on group dynamics of physical activity and inactivity [30]. The results showed that physical activity is hindered and promoted by several social mechanisms, such as age-related social group norms and behaviours, so called (in) activity anchors, social contagion effects, and the way people interact in groups.

In this article, we analyse the quantitative observational data of this study. In this regard, our main focus lies on the identification of contrast groups, i.e. groups which are particularly physically active or inactive. Furthermore, we want to find out which consistent correlates of physical activity [3], such as age, gender or body appearance (BMI: Body Mass Index), characterise these contrast groups. But we also included further factors like group size or communication behaviour in order to analyse how these may influence the physical activity level of groups.

The present paper is therefore guided by the following research question:

- Which factors characterise particularly active or inactive groups?

According to the expected cultural differences, our study should also give answers to a second question:

- In which regard do socio-cultural settings play a role for the occurrence of particularly physically active and inactive groups?

## Methods

### Aim

The overall goal of this paper is to identify the factors that characterise particularly active and inactive groups in an activity-stimulating leisure environment.

### Study design

This paper presents the quantitative data of a large systematic, multi-method (covert) participant observation. The study was designed following methods proposed by Sedlmeier and Renkewitz [31]. Data was collected via a “*paper-pencil approach*” [cf. 21] for two main reasons: firstly, both research sites (see description of research sites below) were too large in order to be covered by one or two static cameras. Therefore, the observers had to be mobile and be able to change observational focus location. Secondly, with an overt video recording, our observers could have been recognised by the visitors of the leisure settings. Since the main objective was to investigate naturally occurring physical activity behaviour, the paper-pencil approach allowed us to conduct the observation covertly und thus avoid possible reactivity effects.

For the quantitative observations, standardized observational description sheets were employed in order to describe the observed groups in as much detail as possible. In order to answer our research questions, we studied “*discrete behaviours*” by strictly recording visible “*low-inference*” structures, i.e. phenomena that an observer can directly see [32].

### Research settings

In order to study factors that influence the degree of physical activity in small groups, we decided to observe group behaviour in leisure settings that hold as little social, time-related, or factual activity barriers as possible. The settings should therefore not only offer opportunities for being physically active in different degrees of intensity but should also offer other opportunities for spending one’s free time, such as sun-bathing, and having lunch or snack breaks [30]. These conditions are given in open air pools, lakes, and ponds which offer both spaces to rest and to be active. Another criterion for the selection of the two research sites was the appropriate size for an observation: The research site should not be too large because observers may lose track of groups, which is the case in many open air pools, lakes, and ponds [30]. We therefore chose a pond in Hawai’i and an open air pool in Germany which were appropriately sized to easily observe and keep track of groups [30]. Furthermore, both sites were chosen for research-economic reasons. The Pond in Hawai’i was very close to the house where the data collectors in Hawai’i, authors Ansgar Thiel and Syra Thiel, lived during the research period (around 45 km away from the University of Hawai’i at Hilo). The open air pool in Germany is close to the University of Tübingen and was thus easily accessible for data collectors in Germany, authors Hendrik K. Thedinga and Olesia Schweizer.

The first chosen observation site was a natural pond, called ‘Champagne Pond Pool’. The pond is located in Kapoho, Hawai’i, and is approximately 200 m long. On its shore, visitors can play ball and tag games; in the pond itself one can go for various activities such as swimming and diving. It also includes a 50 m long coarse-grained, black lava stones area where visitors can lie and sit. Furthermore, directly across the pond is another area of about 300 square meters in size for visitors to rest and sit down. The site is open to the public and can be used free of any charge. Most visitors go to the pond by jeep or truck since traveling to the pond by foot is a difficult hike [30].

The second chosen site was an artificial open air pool in a German village, Entringen, in the southwest of Germany. The Entringen pool has three separated pools: a large swimming pool (16 × 25 m), a medium-sized pool for children and visitors who cannot swim (12 × 12 m), and a very small and shallow kids pool (6 × 6 m). The area also incorporates a very large lawn for sitting, resting, and sunbathing (approx. 100 × 50 m). Furthermore, there is a playground lawn of equal size. This lawn includes a beach volleyball pitch, two very small football goals and basketball posts. Finally, the pool area offers a playground for children and table tennis tables. The Entringen open air pool is a public pool where visitors pay a very small fee per visit (3 euros for adults/1.50 for children and students) [30].

For images of both research sites please click on the following link: http://www.ifs.uni-tuebingen.de/institut/arbeitsbereiche/sozial-und-gesundheitswissenschaften/forschung/observation/images.html

The following physical activities could be observed frequently (Table 1):

**Table 1:** Physical activities at the observation sites [30]

Champagne Pond Pool and Entringen Pool:
•Swimming (lap swimming as well as play)
•Diving activities (competitive and playful)
•Aqua jogging (usually with swim noodle or inflatable mattress)
•Teasing one another and dunking each other under water, water splashing and water bombs (mostly children and adolescents)
•Treading through pool with long breaks at the edge of pool. (cooling off in water when it is very sunny and hot)
•Ballgames in water/pool or on lawn (passing or shooting off friends)
•Wild, playful running, walking around (mostly young children)
•Slow/moderate walking around/playing with children by parents
•Playful jumping into pool or water
Entringen Pool
•Table-tennis, beach volleyball, football (mostly ball passing)
Champagne Pond Pool
•Snorkelling (usually while swimming slowly, sometimes supported by swim noodle)
•Stand up paddle boarding
•Canoeing
•Surfing and wakeboarding (outside the lava stone wall of pond)

### Data collection and sample

All visitors who came to the pools in groups (at least two or more people) were potential participants for the quantitative part of this observation study. In total, we observed and recorded 907 groups with the groups’ size varying between 2 and 8 members. For more details concerning the characteristics of these groups, please refer to the results section below.

Data was collected by two trained observers in Hawai’i, and two different trained observers in Germany. At each observation site, we initially spent around one week in order to establish relevant categories for the quantitative description sheets. The level of participation of the observers was moderate (when sitting at the beach at the Champagne Pond or on benches at Entringen Pool) to active (when swimming in pool or walking around while observing). Once all categories for description sheets were developed, we defined standards and started with a specific observer training, developed by author Ansgar Thiel. Firstly, the observers had to learn about the specifics of the systematic observational method and our research project at large [30]. Secondly, they were instructed on what McKenzie and van der Mars [21] call “*ethical issues, the need for objectivity maintaining confidentiality, and observer etiquette*” [21]. Thirdly, trainees were then introduced to all categories used in description sheets and had to learn how to assess and record categories correctly.

With regards to body shape and age, trainees had to train with BMI body shape-charts and photographs in order to correctly assess BMI and age of depicted people [30].

Training protocol also included a one-week period of explorative observation (with daily visits of location). During this time observers received “*live field-based practice*” [21] by author Ansgar Thiel before the recording started [30]. Furthermore, at both research sites, observers regularly compared individual assessments throughout the data-collection periods in order to achieve a high inter-observer agreement level.

To check the reliability of observation, we executed post hoc rating tests (162 photographs) with the observers. In this test, the observers had to assign the photographs to the body shape and age categories employed for the description sheets (body shape categories: 1. ‘athletic’, 2. ‘normal weight’, 3. ‘obese’; age categories: 1. ‘children’, 2. ‘adolescents’, 3. ‘young adult’, 4. ‘middle-aged adult’, 5. ‘senior’). The observers also had to assess exact BMI and age. The results (Table 2) of the rating tests show a high reliability of the observations.

**Table 2:** Observer rating reliability test [30]

Sample N	*N* = 162
Images n excluded	*n* = 5 (bad quality)
Sample n used for rating	*n* = 157
Mean (M) age in years	M = 30.38 years
Mean BMI	M = 22.73 kg/m^2^
Total ratings by all observers:	471 total ratings
Rating results:	
Age category correct (in percent)	96.60%
Age category errors (n total)	*n* = 16
Age category error rate (in percent)	3.40%
Average deviation from correct age (in years)	2.83 years
Body shape category correct (in percent)	97.24%
Body shape category errors (n total)	*n* = 13
Body shape category error rate (in percent)	2.76%
Average deviation from correct BMI (in kg/m^2^)	1.38 kg/m^2^

The (recorded) observation at the Champagne Pond took place on 105 days from August 1st 2012 to January 1st 2013; and at the Entringen Pool on 51 days from July 1st to September 15th 2014. The observation period was longer and the season slightly different in Hawai’i due to climate conditions: Hawai’i’s climate allows for a significantly longer observation period. There were comfortable temperatures to attend the Pond in Hawai’i during most of the time between August and January; in Germany, in contrast, open air swimming pools are usually only opened in late spring and summer. In the time-period of our observations in Germany, there had been many rainy and cold days, when the temperatures were not comfortable enough for visitors to stay at the site. As a result, we had significantly less observation days in Germany [30].

When observation was possible at the Champagne Pond or Entringen Pool, observers spent between one to four hours at the research site. Reasons for concluding an observation included weather conditions, time restraints, or data saturation in the given situation [30].

### Observation categories

The observation sheets contained 16 categories. These categories covered detailed descriptions about observation site, day category (weekday/weekend), group size, age-related composition of the group (based on age-assessment of observed individuals in groups). Furthermore, gender-related composition of the group, ethnicity-related composition of the group, physical activity behaviour, body shape-related composition of the group (based on body shape-assessment of observed individuals in groups), verbal communication patterns, additional equipment brought to pool, areas of movement, eating patterns, drinking patterns, and smoking behaviour. Low-inference structures which had to be defined before the data collection, such as observed physical activity level and duration, were categorized by using rating scales.

Out of the 16 categories/items that were established for the quantitative observation, five were not included in the data analysis: ethnicity of group members, areas of movement, eating patterns, drinking patterns, and smoking behaviour. We excluded ‘ethnicity of group member’ because of the difficulty to reliably assess this aspect. Furthermore, we decided to exclude the category ‘area of movement‘ since it overlapped with the dependent variable ‘physical activity level of group’. We also had to exclude ‘eating patterns’ and ‘drinking patterns’ as independent variables because we could not be sure whether the observations were valid due to observational limits (distance of the observer, different observation times). Smoking behaviour was not included in the statistical analyses because the contrasting cells were partly too small. For the statistical calculations, we constructed a further category by assigning the data to three group size levels (group size category). The following categories were included in the statistical analyses (Table 3).

**Table 3:** Categories for statistical analyses

Target Variable	Type	Description
Physical activity level of group	Rating scale	1) ‘Very active’ (high intensity activities, such as fast swimming of lanes, playing football/soccer, beach volleyball, ballgames, wild playful running and playing tag games), 2) ‘Moderately active’ (low to moderate intensity activities, such moderate/slow swimming of lanes, water games, playful swimming or diving activities with prolonged breaks, relaxed playing of table-tennis) 3) ‘Rather passive, sometimes in the water’ (sitting around or sunbathing with episodes of standing in the water or being active for short periods of time) 4) ‘Extremely passive’ (lying, sunbathing, sitting in circles on lawn/around pool/shore/on trucks with no active episodes)
Independent Variable	Type	Description
Gender-related group composition	Nominal	Male only, female only, mixed
Observational setting	Nominal	Hawai’i Champagne Pond, Germany Entringen Pool
Age categories	Nominal	Children (0–12 years), adolescents (13–17 years), young adulthood (18–34 years), middle adulthood (35–60 years), seniors (> 60 years)
Age -related group composition (Specific)	Nominal	Adults only, mixed, children only, adolescents only
Age-related group composition (Homogeneous/ heterogeneous)	Nominal	Homogeneous age group (categories see above), mixed-aged group (combinations of different categories)
Body shape categories	Nominal	1) Obese (clearly obese with a BMI over 30) 2) Normal weight (lean/normal weight/slightly overweight with a BMI between 18 and 29) 3) Athletic (BMI over 25, but very muscular with low body fat)
Body shape-related group composition	Nominal	1) Mainly (at least 80%) athletic 2) Mainly (at least 80%) normal weight 3) Mixed (combination of normal weight/athletic with obese) 4) Mainly (at least 80%) obese
Group size	Ratio	Total number of individuals in one observed group (for general statistics)
Group size categories	Ordinal	1) Two or three members 2) Four or five members 3) Six or seven members or more
Additional equipment	Nominal	Did a group have equipment such as chairs, sun loungers, barbeques, cooling boxes, sunshades, etc. or not
Communication level within group	Rating scale	1) Frequent, lively verbal communication between most group members, 2) Occasional verbal communication, 3) Almost no verbal communication among group members
Day of observation	Nominal	1) Workday 2) Weekend

### Statistical analyses

As a basic, explorative analysis, we firstly calculated general statistics in order to assess the significance of differences regarding the degree of physical activity dependent on the target variables. Secondly, we calculated one-way ANOVA (analysis of variance) analyses and t-tests respectively for independent groups. For this, we employed a Bonferroni correction to counteract the problem of incorrectly rejecting a null hypothesis due to calculating multiple comparisons. To check the practical relevance of differences, we calculated the effect sizes of differences using η^2^ (part. Eta squared) and Cohen’s d. Thirdly, we did a classification tree analysis in order to identify contrast groups regarding physical activity and inactivity.

We used classification tree analysis because it “*allows for exploratory identification of contrast groups*” [33] based on potential influencing factors. Classification tree analysis enables furthermore to test several variables for possible interactions effects. Recommended by several researchers [34–37], this analysis method represents an appropriate statistical analytical tool for an explorative analysis of a medium to large data set because it is able to handle both a “*simultaneous treatment of interactions among independent variables*” [33] and manage “*a variety of variable types simultaneously (continuous, ordinal, or nominal)*” [33].

## Results

In total we observed and recorded 907 groups with the groups’ size varying between 2 and 8 members (group size mean = 3.51, SD (standard deviation) = 1.348). 86.1% of the groups were age-homogeneous, 13.9% age mixed. 71% of the groups were categorized as adult groups, 20.9% adult groups with children and/or adolescents, 3.7% children only groups, and 4.2% adolescent only groups. 22.9% were groups consisting of female members only, 12.2% as male only groups, and 64.8% as gender mixed groups.

### General statistics

Our general statistical analysis (Table 4) shows that – on average – the observed groups in the two cultural settings did not differ regarding their physical activity level (*p* = 0.381). All other variables show significant differences. Male groups (M (mean) = 1.70) were significantly more physically active than both female (M = 2.70) and mixed groups (M = 2.87). According to the effect size (part. Eta squared 0.119), the difference is medium to strong.

**Table 4:** General Statistics of main independent variables

Independent Variable	N	Physical Activity Level (M: mean)	SD	Sig.^a^	Effect size
Observational setting					
Entringen Pool	328	2.57	1.032	.381 (n.s.)	0.062 (Cohen’s d)
Champagne Pond	579	2.64	1.192		
Gender-related group composition					
Female	208	2.39	0.734	.000	0.119 (part. Eta squared)
Male	111	1.70	1.014		
Mixed	588	2.87	1.172		
Group size (categories)					
2–3 members (1)	489	2.15	0.962	.000	0.119 (part. Eta squared)
4–5 members (2)	316	3.08	1.177		
6 and more members (3)	102	3.39	0.706		
Age -related group composition (specific)					
Adults only	644	2.49	1.145	.000	0.095 (part. Eta squared)
Age-mixed	190	3.25	1.006		
Children	34	1.85	0.436		
Adolescents	38	2.24	0.675		
Age-related group composition (homogeneous/ heterogeneous)					
Age-homogeneous	781	2.45	1.104	.000	1.236 (Cohen’s d)
Age-heterogeneous	126	3.62	0.757		
Body shape-related group composition					
Mainly Athletic	69	1.35	0.855	.000	0.259 (part. Eta squared)
Mainly Normal-weight	408	2.27	1.135		
Mixed	252	2.96	0.815		
Mainly Obese	178	3.42	0.848		
Additional equipment					
Yes	474	3.12	1.012	.000	1.053 (Cohen’s d)
No	433	2.06	1.001		
Communication level within group					
1 (frequent and lively)	398	3.25	0.880	.000	0.303 (part. Eta squared)
2 (occasional)	346	2.36	1.009		
3 (almost none)	161	1.60	0.990		
Day of observation					
Workday	474	2.46	1.124	.000	- 0.293 (Cohen’s d)
Weekend	433	2.79	1.127		

*n.s* Not significant

^a^Significance level after Bonferroni correction: *p* = 0.05/24 = 0.00208

Our data furthermore strongly suggests that with increasing group size the physical activity level significantly decreases. In other words, the larger a group was, the less physically active it tended to be. The effect is medium to strong (part. Eta squared 0.119).

The age-related composition of the group also plays a relevant role regarding the group’s activity level. Of all recorded age-related group compositions, groups consisting of children only (≤ 12 years) were the most active groups (M = 1.85), groups consisting only of adolescents were the second most active groups (M = 2.24). Adult-only groups were less active (M = 2.49) than children and adolescent groups, but had a higher physical activity level than age-mixed groups (M = 3.25). The effect size of these differences is medium (part. Eta squared 0.095). In this regard it has to be noted, however, that the size of contrasting cells differs strongly. While we recorded 644 ‘adults only’ and 190 ‘adults with children’ groups, we observed only 34 children only and 38 teenager-only groups who were not accompanied by adults during the observation. Comparing age-homogeneous and age-heterogeneous groups, we also found a significant difference. Age-heterogeneous groups are significantly less active (M = 3.62) than age-homogeneous groups (M = 2.45). This effect is very strong (Cohen’s d 1.236).

The data shows a very strong effect for the recorded body shapes of the group members (part. Eta squared 0.259). Groups mainly consisting of clearly obese individuals (M = 3.42) were significantly less active on average than normal weight groups (M = 2.27) and athletic groups (M = 1.35), who had the highest average physical activity level of all groups. In the context of the body shape-related group composition, it is also important to point out that mixed groups were considerably less active with M = 2.96 than normal-weight groups.

We further found that bringing along ‘additional equipment’, such as sun loungers, chairs, or cooling boxes, negatively correlates with the group’s physical activity level. The groups who brought additional equipment to the pool had a significantly lower physical activity of M = 3.12 than the groups who did not have any additional equipment apart from towels (M = 2.06). The effect is very strong (Cohen’s d 1.053).

The statistical analysis shows the strongest difference of physical activity (part. Eta squared 0.303) dependent on the different levels of communication within the observed groups. When there was almost no verbal communication within a group, the average physical activity was very high (M = 1.60). Occasionally communicating groups were significantly less physically active on average (M = 2.36), while groups having very frequent and lively verbal communication had the lowest average physical activity level (M = 3.25).

The explorative statistical analysis (Table 4) only allows to identify singular determinants of physical activity. To analyse interaction effects and differentiate the most distinctive contrast groups regarding physical activity, we calculated a tree analysis model.

### Classification tree analysis

**Fig. 1:**
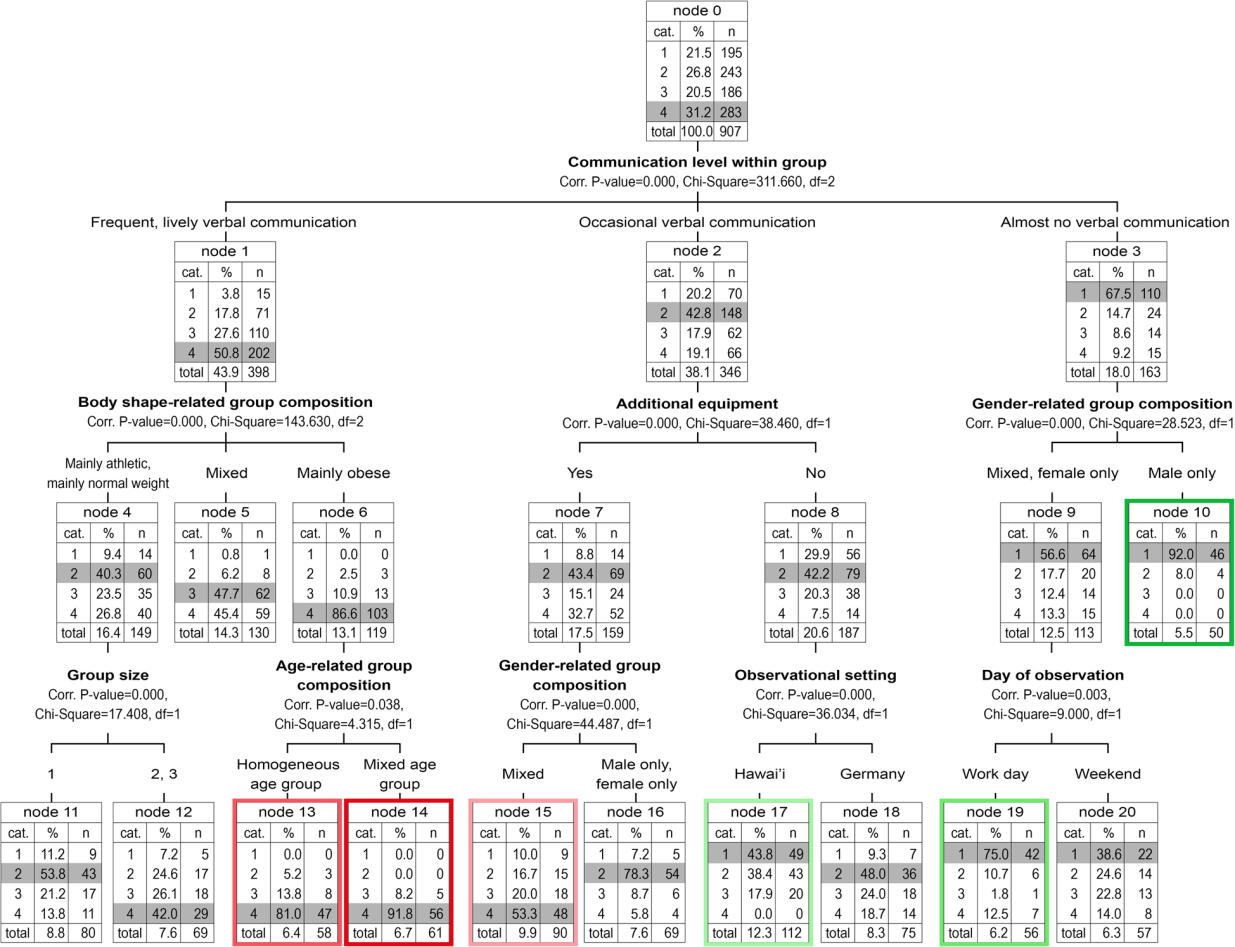
Classification Tree of Predictors associated with Physical Activity. Physical activity level of group: 1 = very active; 2 = moderately active; 3 = rather passive, sometimes in the water; 4 = extremely passive. Contrasting Clusters (Colours): Dark green = highest physical activity level; Green = 2nd highest physical activity level; Light green = 3rd highest physical activity level; Light red = 3rd lowest physical activity level; Red = 2nd lowest physical activity level; Dark red = lowest physical activity level. Group size category: 1 = 2 or 3 members; 2 = 4 or 5 members; 3 = 6 or more members

The tree model calculated is depicted in Fig. 1. For the tree analysis, we left out the variable ‘age-related group composition (specific)’ because the groups consisting only of children, and only of adolescents, had too small cell numbers. Taking a first glance at the tree diagramme, it can be seen that a group’s level of physical activity can be predicted by seven interacting factors on three different levels. On the first level of the classification tree, the factor that explains the largest amount of variance in physical activity is depicted: verbal communication. We described the differences in physical activity depending on the level of communication in the general statistics.

At the second level, the following three factors significantly contrast the physical activity level of subgroups: Body shape–related group composition, additional equipment, and gender. However, these factors only had a significant contrasting impact on the frequently and lively communicating groups. All groups of this sub-cluster had a relatively low average level of PA (physical activity). Yet, in frequently communicating obese groups, who were more or less inactive over the entire period of observation, the hindering effect of verbal communication on physical activity was very strong. Additional equipment is also a significantly contrasting factor, but only for occasionally communicating groups. Finally, gender is a significantly contrasting factor only for those groups which were recorded with almost no verbal communication. While some non-communicating mixed gender and female-only groups were inactive, all male-only groups were at least moderately active for a longer observation period.

At the third level of the tree, the group’s size, the age-related composition of the group, gender, the cultural setting, and day of the observation have contrasting effects in combination with other factors. Small groups with normal weight or athletic members who communicated frequently were significantly more active on average than comparable groups of medium or large size. The age-related composition of a group only has significantly differentiating effects for obese groups, although both age-homogeneous and age-heterogeneous obese groups were by tendency rather inactive. Gender differentiates the level of physical activity on the third node of the tree for occasionally communicating groups who brought additional equipment. In this regard, male-only and female-only groups were significantly more active than mixed gender groups. Occasionally communicating groups who brought no additional equipment were significantly more active than groups who brought equipment. However, this effect was particularly strong in the Hawai’ian setting. The factor of whether visitors came to the pool on a work day or the weekend is only able to differentiate within non-communicating mixed gender or non-communicating female-only groups. While these groups tended to be very active during workdays, a significant amount of these groups were rather inactive on the weekends.

The tree also shows that one important factor that appears to foster physical inactivity is the attribute ‘mixed’. With regard to body shape (node 5), age composition (node 14), and gender (nodes 15 and 9), the groups that were ‘mixed’ in the respective categories, had a lower physical activity level. For instance, on level 2, the groups that included both normal weight as well as overweight individuals (node 5) were more inactive than the groups which consisted of only athletic and normal weight members. The same effect can be seen for age: on the third level of the tree, the frequently communicating, mainly obese groups which included individuals of mixed age were more inactive that those of homogeneous age composition. Finally, groups which consisted of both female and male members tended to be more inactive than female or male only groups (see nodes 15 and 9). In fact, the latter two factors (mixed age in node 14 and mixed gender in node 15) characterise – together with other factors - the most inactive groups overall.

Looking at the twelve terminal nodes of the tree (nodes 5, 10, and 11–20), we identified six so called ‘contrasting clusters’ with regard to physical activity. Although the correct statistical term is ‘contrast group’, we will refer to them as ‘contrasting clusters’ instead in the following. This is because we are dealing with ‘groups’ of people in the data, and we would like to avoid confusion. These contrasting clusters will be briefly described in the following:

### Contrasting clusters concerning physical activity

1. The contrasting cluster with the highest physical activity level on average incorporates groups that had almost no verbal communication and consisted of male members only (5.5% of all groups observed). 92% of the groups belonging to this cluster were assessed with the highest physical activity level during their stay (node 10 of tree).
2. The contrasting cluster with the second highest physical activity level on average (6.2% of all groups) includes non-communicating groups with mixed or female-only gender composition that visited to the pools on workdays. 75% of the groups belonging to this cluster were recorded with the highest physical activity level, and 10.7% were assessed as moderately active (node 19).
3. The contrasting cluster with the third highest physical activity level (12.6% of all groups observed) comprises occasionally communicating groups in Hawai’i who did not bring additional equipment to the site. In this cluster, 43.8% were very active, another 38.4% were moderately active during the observation periods (node 17).
4. The contrasting cluster with the third lowest physical activity level includes occasionally communicating groups of mixed gender that brought additional equipment to the pool (9.9% of all groups). More than half of these groups (53.3%) were extremely passive, additional 20% were ‘rather passive’. 16.7% of this cluster were recorded as moderately, only 10% as vigorously active (node 15).
5. The contrasting cluster with the second lowest physical activity level on average consists of frequently communicating, age-homogeneous obese groups (6.4% of all groups observed). In this cluster, 81% were extremely passive, another 13.8% rather passive (i.e. sitting around or sunbathing with episodes of standing in the water or being active for short periods of time) (node 13).
6. The contrasting cluster with the lowest physical activity level on average includes frequently communicating age-heterogeneous obese groups (6.7% of all groups). Almost all groups of this cluster (91.8%) were extremely passive, and the remaining 8.2% were recorded with the second lowest physical activity as rather passive with only short periods of low-level activity (node 14).

## Discussion

The main goal of our mixed-method observational study was to investigate social determinants of physical activity of groups in two leisure settings in Hawai’i and Germany [30]. This article presents the analysis of the quantitative observational data of this study with the main focus being laid on the identification of contrast groups, i.e. groups that are especially active or inactive. In this regard, the analysis was guided by two questions: which factors characterise extremely active or inactive groups? And, in which regard do the two different socio-cultural settings have an impact on the occurrence of such active and inactive groups?

Before discussing the results, we would like to discuss limitations of our findings. We addressed some of these already in the qualitative article of our mixed method observational study [30]. Firstly, the observational study is only able to give an account of visitors’ activities and behaviours during their stay at the pools. In order to stay covert, we did not speak to any of the visitors. Therefore, we were not able to collect data about the physical activity behaviour of participants outside the research setting and the observational period. By the same token, we do not have information about participants’ purpose or motivation for attending the research site. Consequently, we cannot rule out that some of our results could be explained by the participants’ intents for going to the pools [30]. The fact that smaller sized groups were more active than larger groups, for example, may be a result of motivational differences. Hence, while some larger groups perhaps come to the pool with the intention of relaxing and socialising joyfully together, some groups of pairs possibly visit the pools with the explicit motivation to swim lanes. An additional limitation of the study results from the fact that we executed the observation covertly. In order to stay unnoticed by participants, we sometimes had to record and complete description sheets for observed groups outside the observational areas. Observers had to memorize their observations for a short time in these cases. This was especially the case for the pond in Hawai’i, where several observations were executed from the water. Although we trained and tested memorizing the observations, observational errors may have occurred in these cases [30]. Finally, we should point out that we had different amounts of observational days at the research sites and more data for the Hawaiian site due to different climate and weather. This may have affected the estimates. However, since we used all possible days to collect data in Germany, the collected data should be representative for the entire season of that year.

With regards to the main research questions, our findings partially confirm previous studies. The cultural setting itself did overall not appear to have a very significant impact on the occurrence of physically very active or very inactive groups. This lack of major differences may appear surprising. Yet, it indicates that physical activity in leisure settings is significantly less influenced by cultural values than, for example, by the way in which individuals perceive and present their body [30]. However, one minor difference should be highlighted. The tree analysis showed that occasionally communicating groups that brought no additional equipment were more active in Hawai’i than in Germany. This difference may stem from a cultural difference which data collectors noticed in Hawai’i. In Hawai’i, people who spend their leisure time with others at the beach generally tend to ‘domesticate’ their site heavily with additional equipment [30]. However, in Germany, relaxing with others in leisure settings is not necessarily coupled with ‘furnishing’ the environment in a cosy way. This could also be the reason that the difference in the ways of spending ‘social’ sedentary time and ‘functional’ exercise was more distinctly observable in Hawai’i than in Germany [30]. To our knowledge, there are no studies which explain such cultural differences in spending leisure time in groups and its potential impact on physical activity. However, this may also be a result of research settings’ characteristics - we discuss this in more detail below regarding the results about additional equipment.

Although the cultural setting did not have a direct influence on the occurrence of activity, our study is mostly consistent with previous research showing that the social environment in general and the embeddedness in a social network in particular have a very relevant impact on a person’s physical activity level [10–13, 15, 38]. Our results furthermore strengthen the hypothesis that specific group structures play a significant role regarding the activity of the group members [18]. For example, sitting together in groups and chatting, appears to be hardly compatible with physical activity for adults. In this regard, the ‘furnishing’ of the group’s site with additional equipment [30] seems to additionally reduce the willingness to be active. In contrast to adult groups, the high activity level of children-only groups shows that for them, playful, boisterous physical activity with lively verbal and non-verbal communication may be a relevant strategy of collective bonding [30].

Overall, the single most determining factor for activity/inactivity is the body appearance of group’s members. While obese groups had the lowest average activity level, athletic groups were the most active. The fact that obesity turned out to be one of the strongest activity limiting factors confirms previous epidemiological studies [3]. The observation that mixed-BMI groups were significantly less active than normal weight or athletic groups could be regarded as a social contagion effect, i.e. the spread of a low degree of physical fitness. This is consistent with findings by Carrell et al. [14] that it is especially ‘poor fitness’ and unhealthy behaviours that appear to spread in social networks. Christakis and Fowler suggested that such a spread of behaviours may be explained by ‘mirroring’ [39, 40]. A recently published work by Datar and Nicosia explains in this regard that “*mirroring is more commonly observed in friends and family networks, peer networks, and cultural groups, where individuals may mirror the behaviour of significant others or those they esteem*” [40]. This effect supports Turner’s theory on group behaviour: when individuals become member of a social group, it impacts their behaviour because they perceive other group member’s behaviour and this influences how they act themselves [19]. In this context, we should highlight that not all overweight visitors were inactive in general. In fact, our observational notes indicate that we did observe some obese visitors who were very active. Yet, they were often on their own and not part of groups, and, thus, not part of the analysis.

Also, the level of communication within a group was a very strongly activity influencing factor. Frequently communicating groups were clearly less active than non-communicating groups who had the second highest average level of activity. In our study on the qualitative observations [30], we provided a possible explanation of this finding in detail. We assume that the activity-hindering effect of lively communication can be attributed to the phenomenon that holding an interacting adult group together requires ‘physical discipline’, which means that members of lively communicating groups cannot come and go whenever they want to.

A closely related important factor that characterises inactive groups was a large group size. Our general statistics strongly suggests that with increasing size, the activity decreased. However, regarding the contrasting clusters, the tree analysis showed that group size had a differentiating effect only in mainly athletic and normal weight groups with a high level of verbal communication. Referring to the point raised above and our qualitative study this could be explained by the phenomenon that in large, vividly communicating groups, the ‘social norm’ is to be physically inactive in order to socialise and to not disturb the group’s conversation [30]. According to Turner, such group behavioural rules and norms play an important part in group members’ behaviour [19]. Since athletic and normal weight groups generally tended to be more physically active, this effect may have been stronger than in groups of obese people. The result that a large group size presents a barrier to activity also supports findings by Kilpatrick that physical activity and exercise is often viewed and executed as an individual activity [30, 41].

Furthermore, the gender-related composition of a group had a strong effect on physical activity. While mixed groups tend to be rather inactive, male only groups had the third highest activity level on average.

The finding that groups coming to the beach only with a towel are more active than groups bringing barbecue grills, sun-lounges, chairs etc. can be regarded as an expression of leisure motives. Bringing along additional equipment speaks for the motive of ‘domesticating’ one’s leisure setting, while the absence of additional equipment could be interpreted by a functional orientation of the visitors [30]. The observation that the absence of additional equipment is stronger correlated with the degree of a group’s physical activity in Kapoho/Hawai’i than in Entringen/Germany could be explained by the recreational nature of the observed leisure site. Without chairs, the black lava beach in Kapoho was not as convenient for a long stay in an inactive position as the lawn in Entringen. We therefore assume that in Kapoho, people who came to the beach without trucks, barbecues, chairs and sun shades may have had the primary intention to be physically active during their stay. This may have been the reason why they did not need the additional equipment.

However, our classification tree analysis shows that the level of physical activity in groups is to a relevant degree the result of a combination of different factors. This confirms the results of a friendship network analysis of obese persons’ health behaviours by De la Haye and colleagues [13] who also came to the conclusion that several factors influence health behaviour outcomes. In our study, for example, obesity turned out to be not a barrier per se; it rather hinders activity particularly under specific circumstances. According to the results of the tree analysis, such a factor is frequent and lively verbal communication. It has a particularly hindering effect on the physical activity of obese groups. Lack of verbal communication, on the other hand, is mainly a characteristic of very active groups. However, a gender effect became apparent in this regard, namely, that non-communicating male groups were significantly more active than non-communicating female groups. In fact, this group configuration was one the contrast clusters with highest physical activity level of all. We thus assume that non-communicating male groups came to the pool very often with the purpose of being physically active or doing sports. In contrast to this, in non-communicating female and mixed-gender groups, the level of activity was much higher on weekdays than on the weekends. This suggests that the degree to which these groups allow themselves to relax correlates with the amount of possible free time. The observed gender difference could be explained by the observation of Kilpatrick et al. that particularly men are “*inclined to view exercise and fitness activities as an opportunity to pursue and achieve ego-related outcomes*” [41]. Against this background, one could assume that physically active men who spend their free time together have a rather functional understanding of leisure activities.

Finally, it is important to highlight that ‘mixed’ groups regarding gender, body shape, and age tend to be more inactive than homogeneous groups. Hence, physical activity seems to decrease when people of different body size, gender, and age socialise. This could - again - be a special effect of social contagion in the sense that active persons mirror the behaviours of the inactive [14, 39, 40]. In this regard, the reduction of activity in mixed groups could be seen as a ‘courtesy’ or ‘protection’ habit [30]. A central role in this respect play so called ‘inactivity anchors’ [30]: for example ‘overprotective parents’ who limit their children’s activity behaviour or young individuals who limit their own activity out of consideration of the older group members. The same could be assumed about the interaction of athletic/normal weight persons with obese persons or for the interaction of gender. This habit was brought to the attention of data collectors when they discussed observations with locals in both settings [30]. When people of different age, body shape, and gender socialise, the more active individuals may adjust their behaviour as a courtesy to the rest of the group.

## Conclusion

Our study shows that a number of social factors determine active and inactive groups. Future health promotion strategies should therefore consider these determinants in group level interventions instead of just focusing on individual motivational approaches [38, 42].

Our findings contradict somewhat the general health scientific assumption that the inclusion in a social network is one of the most beneficial health factors [43]. Although joyfully chatting with others in a relaxing environment is an important element of health promotion programmes, our results show that it can also hinder physical activity, at least in adults. To overcome this ‘health promotion paradox’, future research has to investigate how the ‘socially connecting’ effects of joyful play activities can be transferred into health related exercise programmes. A potential strategy could be to use team sports as a medium of health promotion because they are seen as more motivating than individually executed exercise and “*may facilitate improved adherence to physical activity*” [41], particularly because of their joyful and playful nature. A very important target group in this regard are obese people. Lecturing obese people about their potential health hazards often has counterintuitive effects on their weight management and health behaviour: they tend to overeat, avoid diets and engage in less physical activity particularly if they have been victims of weight-related stigmatization [44–47]. Finding ways to create joyful environments which foster physical activities of obese people, without lecturing or humiliating them, is therefore a very important challenge for health research.

For future research, we recommend to employ the method of participant observation in order to study physical activity in other social settings. In this context, it may be beneficial to also include research settings such as parks, beaches, or playgrounds [30], but also educational contexts or workplace environments.

## Additional files

Additional file 1 Observation-Data-File. (SAV 25 kb)

Additional file 2 Readme-File for Observation Data. (DOCX 18 kb)

## Abbreviations

ANOVA: Analysis of variance
BMI: Body mass index
M: Mean
n.s.: Not significant
PA: Physical activity
SD: Standard deviation
