# Supplementary material for: Why are some groups physically active and others not? A contrast group analysis in leisure settings
Source: BMC Public Health. 2018 Mar 20;18:377. doi: 10.1186/s12889-018-5283-2 (PMC5859510; doi:10.1186/s12889-018-5283-2)
Supplement: Supplementary file 2 — Readme-File for Observation Data. (DOCX 18 kb) [file 12889_2018_5283_MOESM2_ESM.docx]

**ReadMe File for SPSS data file**

The provided SPSS data file represents the data of all variables used for the analysis for the research article “…..” published in “…..”

Data was collected with standardised description sheets. After data collection, data was transferred into excel sheet and then transferred into an SPSS file.

The explanations for each variable are provided in the article. We also provide a short description in the following. Furthermore, the values for each items are listed for each category.

**Explanation of Variables with value meanings:**

1 = Case number overall: assigned case numbers to data set

2 = Case number setting: assigned case number to data for each setting

Important to note here: “Case numbers”: These case numbers represent the order in which our original excel spreadsheets contains the data. The case numbers do not reflect the chronological order of when the data was collected. This is due to data collection proceedings and data transfer into excel and SPSS files.

3 = Observational setting: setting where data was recorded: Champagne Pond Pool, Hawaii or Entringen Pool, Germany

Value: 1 = Germany

2 = Hawaii

4 = Day of observation: On which day of the week was data recorded: weekend or workday

Value: 1 = Weekend

2 = workday

5 = Physical activity of group: Observed physical activity of group

Value: 1 = very active

2 = moderately active

3 = rather passive, sometimes in the water

4 = extremely passive

6 = Body shape-related group composition: Observed composition of body shapes of group

Value: 1 = mainly athletic

2 = mainly normal-weight

3 = mixed

4 = mainly obese

7 = Age-related group composition (specific): Specific age categories observed within a group

Value: 1 = adults only

2 = adults with children/adolescents

3 = children only

4 = adolescents only

8 = Age-related group composition (homogeneous vs. mixed): Did all members of a group belong to one age category (homogeneous) or to different ones (mixed)

Value: 1 = homogeneous

2 = mixed

9 = Gender-related group composition: Observed gender composition of group

Value: 1 = female only

2 = male only

3 = mixed

10 = Group size category: Observed size of group in categories (group size ratio assigned to three group size levels)

Value: 1 = 2 or 3 members

2 = 4 or 5 members

3 = 6 or more members

11 = Group size ratio: Observed size of group in ratio (actual size in numbers)

12 = Communication level within group: observed verbal communication patterns within a group

Value: 1 = frequent and lively verbal communication

2 = occasional verbal communication

3 = almost no verbal communication

13 = Additional equipment: Did the recorded group bring additional equipment to the site (such as sun loungers, chairs, cooling boxes)

Value: 1 = yes

2 = no

All calculations were performed with SPSS Statistics Version 23

For the general statistics, we performed means, standard deviations, frequencies, one-way Anova, t-tests for independent groups, effect sizes (Cohen’s d, part. Eta squared)

For the classification tree analysis, we performed Chi-square Automatic Interaction Detectors (CHAID).
